# Creating and Keeping Exercise Gains Into Your 70s, 80s and 90s

**DOI:** 10.1093/geroni/igaa057.2788

**Published:** 2020-12-16

**Authors:** Kenneth Manning, Teresa Kopp, Kris ann Oursler, Michelle McDonald, Miriam Morey, Katherine Hall, Cathy Lee, Jamie Giffuni

**Affiliations:** 1 Durham VA Health Care System, Durham, North Carolina, United States; 2 Canandaigua VA Medical Center, Canandaigua, New York, United States; 3 Salem VA Medical Center, Salem, Virginia, United States; 4 VA Pacific Island Healthcare System, Honolulu, Hawaii, United States; 5 Veterans Health Administration, Durham, North Carolina, United States; 6 Duke University, Veterans Health Administration, Durham, North Carolina, United States; 7 VA Greater Los Angeles, Los Angeles, California, United States; 8 Baltimore VA Healthcare System, Baltimore, Maryland, United States

## Abstract

Background: Gerofit, an exercise program for older Veterans, is undergoing national dissemination (17 sites in 6 years). Four sites have accrued 4-year functional outcomes (gait speed, 8-foot-up-and-go, 30-second chair stand, and six-minute walk). Methods: Functional assessments were administered quarterly in first year and annually thereafter. Individuals with baseline and at least two follow up measures were included for analysis (n=587). Means were gathered across each timepoint. Results: Mean values for functional assessments from baseline to 4 year were as follows: gait speed m/s- 1.04, 1.12, 1.13, 1.13, 1.09, 1.07, 1.13; 8 ft-up-and-go seconds- 7.6, 6.82, 6.69, 6.69, 7.29, 7.29, 7.51; 30 second chair stands 11.88, 14.06, 14.72, 14.89, 14.69, 14.71, 14.96; and six-minute-walk yards- 499, 532, 541, 544, 531, 530, 556. All follow up measures were significantly improved over baseline (P<.01) and superior to normative age-related decline. Implications: Results indicate that exercise promotes compression of morbidity and improved functional health.

